# Quantitative Description of Isomorphism in the Series of Simple Compounds

**DOI:** 10.3390/ijms241411324

**Published:** 2023-07-11

**Authors:** Andrzej Kuczumow, Mieczysław Gorzelak, Jakub Kosiński, Agnieszka Lasota, Anna Szabelska, Tomasz Blicharski, Jacek Gągała, Jolanta Wawrzyniak, Maciej Jarzębski, Mirosław Jabłoński

**Affiliations:** 1 Lab 196, Radawiec Duży 196, 21-030 Motycz, Poland; andrzej.kuczumow@gmail.com; 2 Department of Orthopedics and Rehabilitation, Medical University of Lublin, 20-059 Lublin, Poland; b.leszczynska@umlub.pl (M.G.); kuba.kosinski@gmail.com (J.K.); tomasz.blicharski@umlub.pl (T.B.); miroslaw.jablonski@umlub.pl (M.J.); 3 Chair and Department of Jaw Orthopedics, Medical University of Lublin, Chodźki 6, 20-093 Lublin, Poland; 4 Department of Dental Techniques with the Lab of Modern Technologies, Medical University of Lublin, Chodźki 6, 20-093 Lublin, Poland; anna.szabelska@umlub.pl; 5 Department of Orthopedics and Traumatology, Medical University of Lublin, K. Jaczewskiego 8, 20-090 Lublin, Poland; jacekgagala@gmail.com; 6 Faculty of Food Science and Nutrition, Poznań University of Life Sciences, 60-624 Poznań, Poland; jolanta.wawrzyniak@up.poznan.pl; 7 Department of Physics and Biophysics, Poznań University of Life Science, 60-637 Poznań, Poland

**Keywords:** isomorphic series, X-ray diffraction, energy of crystallographic transformations, apatites, change in universal crystallographic dimension

## Abstract

The introduction of the notion of energy change resulting from the ion exchange in apatites leads to the question: how can some simple isomorphic series be described using the mentioned idea? We concentrated on the simple isomorphic series of compounds: apatite, bioapatite, calcite, aragonite, celestine, K-, Zn- and Cu-Tutton’s salts. It was demonstrated in all the series, except Tutton’s salts, that the change in energy and the change in the crystal cell volume are, in a simple way, dependent on the change in the ionic radii of the introduced ions. The linear relationships between the variations in energy and in the universal crystallographic dimension d were derived from the earlier equations and proven based on available data. In many cases, except the Tutton’s salts, linear dependence was discovered between the change in energy and the sinus of universal angle Θ, corresponding to the change in momentum transfer. In the same cases, linear dependencies were observed between the energy changes and the changes in the volumes of crystallographic cells, and mutually between changes in the crystallographic cell volume V, crystallographic dimension d, and diffraction angle Θ.

## 1. Introduction

Isomorphic compounds, due to their common existence and importance in nature, have attracted a large amount of attention from chemists and mineralogists [1]. The isomorphic chemicals form even the whole series of chemical entities with exchanging some fragments [2], for example, main cations in salts, but without breaking the symmetry of crystallographic cells (diadochy). This suggests the possibility of easy ion exchange between different members of the series. Also, the formation of solid solutions between the members is a characteristic feature [3,4,5,6,7,8]. The inverted problem of the separation of components of solid solutions is also considered [9]. However, the question of isomorphous series is badly quantified in a simple manner. Mitscherlich [10,11] and Goldschmidt’s rules [12,13] are some qualitative indications for the estimation of the ability to form the series. More detailed theoretical considerations are rather complicated [14]. The isomorphic transformations pose great meaning in the world of minerals and biominerals since they explain why the involvement of some cations and anions is allowed while the other ones are not. Moreover, the naturally occurring biominerals are most often supported on apatite, calcite and aragonite, each of them forming the separate isomorphic series. The problem of tailoring the important materials to the demands of users, e.g., the biomaterials applied in dentistry and orthopaedics, can be easier to solve when we can estimate the possibilities of an easy and allowed ion exchange. Of course, biocompatibility plays an important role, but this is another problem, outside the scope of this contribution.

While considering bioapatites, we derived the energetic balances for ionic exchanges in those compounds. The total exchanges of Ca on other cations were considered. Even the full exchanges did not result in the change in the crystallographic class of the compounds. In this sense, one could consider those ion exchanges as the reactions of synthesis of the consecutive members of the isomorphic series.

The main aim of this contribution is to check if our reasoning is valid in the case of typical isomorphic substances, i.e., those derived from the apatite [15], bioapatite, calcite (rhombohedral class), aragonite and celestine (orthorhombic) series and some of Tutton’s salts [16] (monoclinic). Here, we might also treat the next members of the series as if they were formed by the ion exchange of one of always the same ionic component, most often the cation, of the previous member of the series. It is interesting to know whether or not the energy change during this reaction is dependent on the difference of the ionic radii of exchanged cations and on the difference in the universal crystallographic dimension d of considered compounds. The last point is to establish if the isomorphic variabilities obey any simple and universal quantitative rules.

### Theory

In our previous papers [17,18], we considered the question of whether or not it was possible to derive the energy changes from the Braggs’ equation [19] for the case of the ion-exchange transformations of biological apatites. We applied the specific, not wave, but energy-based form of Braggs’ law:

n × 2.4/E = 2d × sinΘ(1)

where E is energy in kiloelectron volts, [keV], d is the universal dimension [Å], and Θ is the angle [deg] between the exciting beam of X-rays and the scattered beam; regarding n, an order of the wavelength was set here as 1. Using the assumptions and transformations shown in papers [17,18], the following expressions for the energy difference were derived:

ΔE = (6.2/d_1_)(1/sinΘ_1_ − 1/sinΘ_2_) (2)

ΔE = (6.2/sinΘ_2_)(1/d_2_ − 1/d_1_) (3)

ΔE = −6.2 × Δd/(d^2^ × sinΘ) (4)

ΔE = −6.2 × ΔsinΘ/(d_2_ × sin^2^Θ_2_) (5)

ΔE = −(1/6.2) × Δd × E^2^ × sinΘ (6)

The results are finally recalculated to electron volts by multiplying by 1000. We can estimate the equivalency of the above equations by a simple check (see Appendix A).

The universal dimension d can be calculated for the (1,1,1) configuration for the systems studied in this contribution from the equations:

Hexagonal (for apatite, calcite):

1/d^2^ = 4 (h^2^ + hk + k^2^)/3a^2^ + l^2^/c^2^ (7)

Orthorhombic (aragonite, celestine):

1/d^2^ = h^2^/a^2^ + k^2^/b^2^ + l^2^/c^2^ (8)

Monoclinic (Tutton’s salts):

1/d^2^ = h^2^/(a^2^ sin^2^ β) + k^2^/b^2^ + l^2^/(c^2^ sin^2^ β) − 2 hl cos β/(ac sin^2^ β) (9)

We know from our experience that only particular compounds can be comparable, when one considers the d quantity from Bragg’s equation. It allows for us to overcome the questions of the variability of compounds. Moreover, only the d dimension is immediately connected with the total energy in Bragg’s equation; other dimensions carry this out in an indirect way. Of course, only the total energy of changes is interesting for us as a value representative for the whole molecule, not this portion, which is connected with the changes along the selected axis.

When the above equations were applied for solving the problem of energy changes during ion exchanges of different cations for Ca^2+^ ion in hydroxyapatite, the next relationships could be detected [17]:

ΔE = 1.125 + 21.11Δr(10)

where r is the ionic radius [20]. This dependence is a linear one. The energy changes also with the variability of the crystallographic cell volume of apatite. Here, the relationship is:

ΔE = 13.37 + 4.965ΔV − 0.0056(ΔV)^2^ (11)

The dependence is nearly linear, but with a small correction of the second order. When one wants to establish the behavior of crystallographic cell volume together with the growth of an ionic radius of an introduced cation, then the following is valid:

ΔV = −4.178 + 4.411Δr + 0.0265(Δr)^2^ (12)

Here, the small correction of the second order is also demanded.

We assume that similar procedures can be applied for the case of at least some isomorphic substances.

## 2. Results

**Figure 1:**
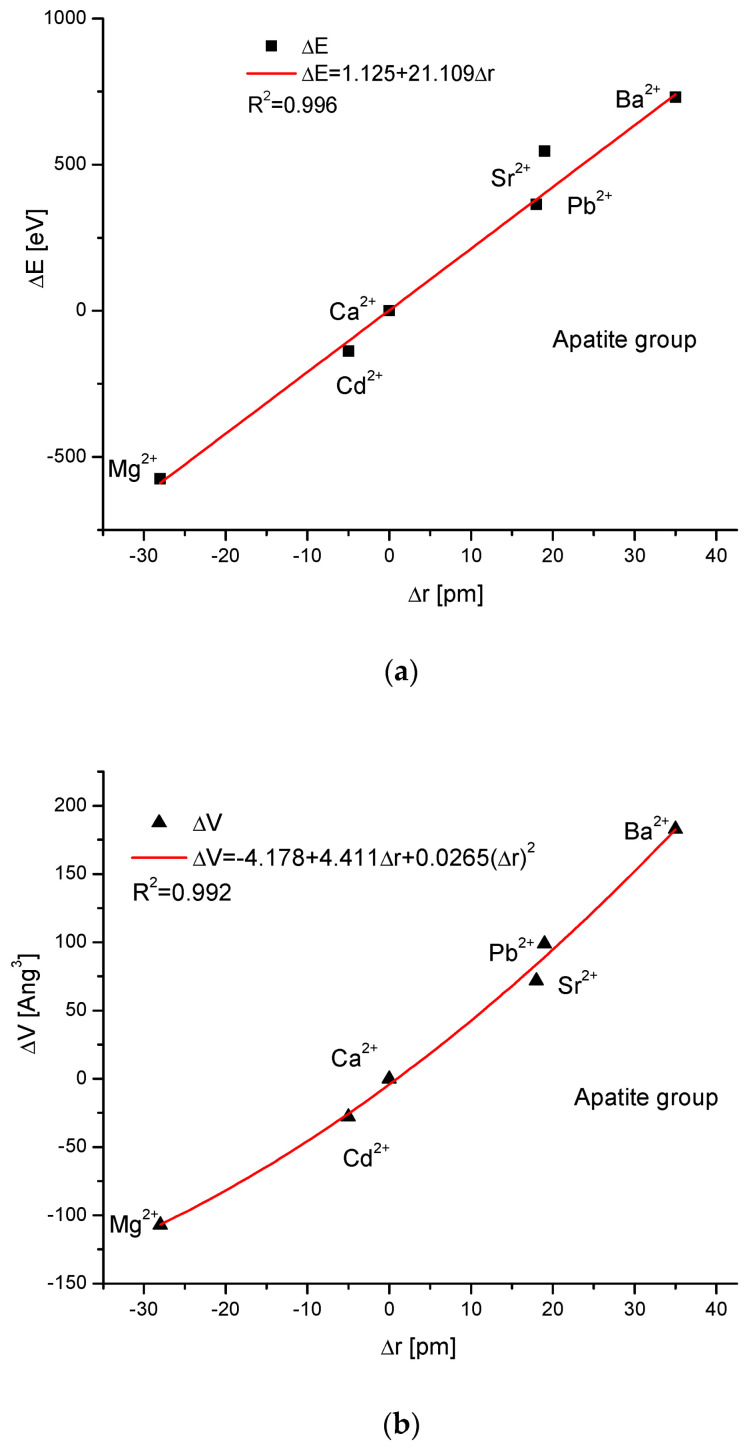

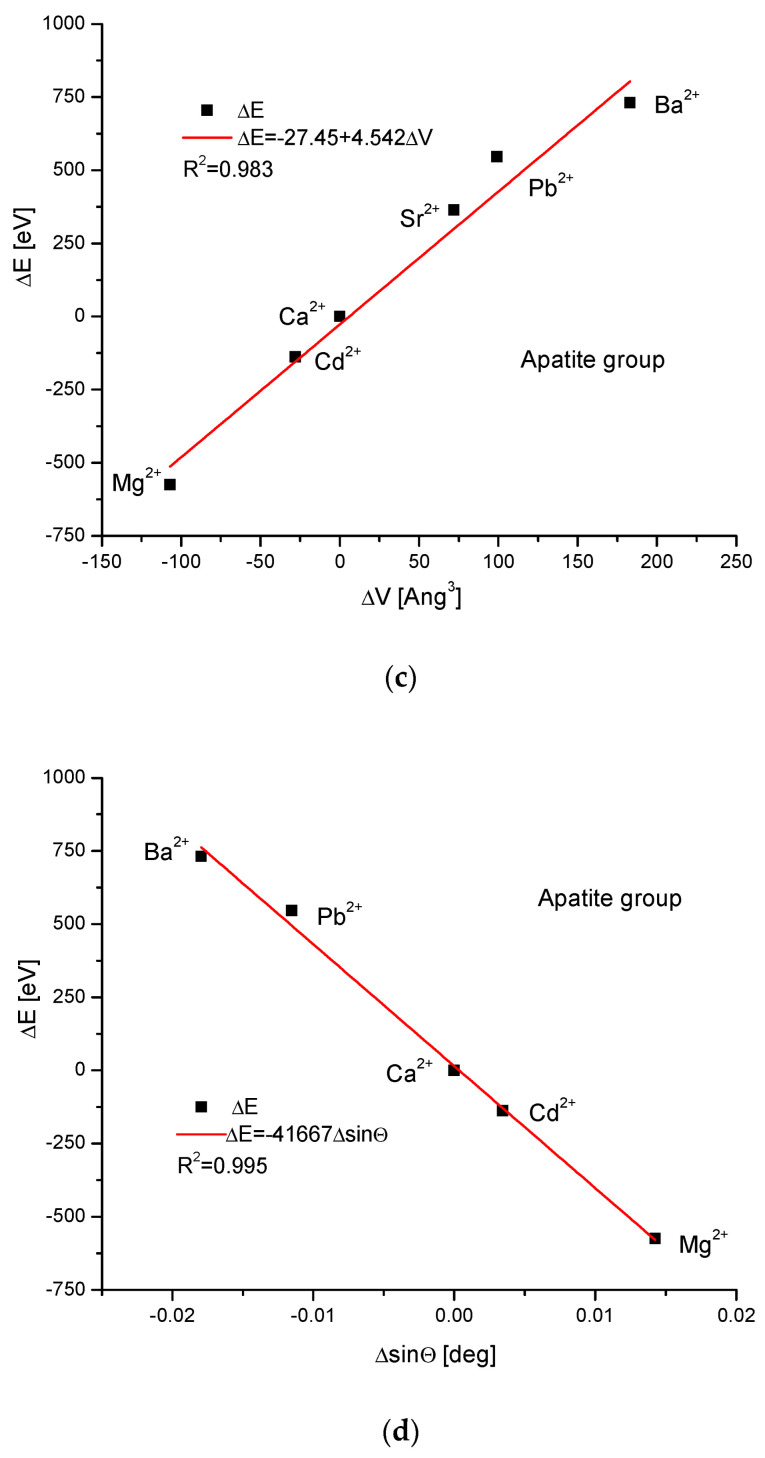

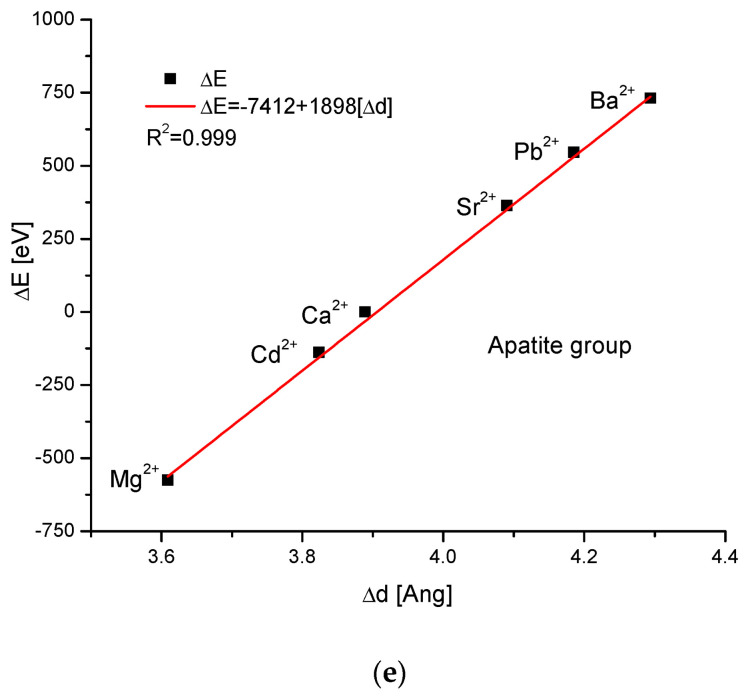
Relationships in apatite isomorphic series between: (**a**) the energy of total ion exchange and the change in ionic radius of exchanged elements; (**b**) the volume change in the crystallographic cell against the change in ionic radius of incoming cations; (**c**) the change in energy corresponding to the change in the volume of the crystallographic cell; (**d**) the changes in energy and sinΘ; (**e**) the influence of the universal crystallographic parameter d on the variation in energy.

As the first one, the apatite system was considered [21], which had been earlier treated in position [17]. Although, essentially, Mg does not form apatites, the wide data on the substitution of Mg into apatites can be found [22]. Figure 1a shows a strict linear dependence between the calculated energy of ion exchanges and the difference in ionic radii of the elements engaged in the process. If we know the crystallographic class of the compound and the relevant distance and angle values of the crystallographic cell, then the calculation of the cellular volume is trivial. The values of volumes as compared to the ionic radii are shown in Figure 1b. The elegant parabolic relationship is obvious, with a small correction of the second order. The dependence of energy change on volume change is strictly linear (Figure 1c). This can also be considered oppositely—it is the energy necessary for the volume change. Next, the correlation between the energy change and the difference in sinus of Θ angle from diffraction measurements was studied, and this correlation was perfectly linear. Finally, the junction between the energy change and the variability of universal crystallographic dimension d was also perfect.

**Figure 2:**
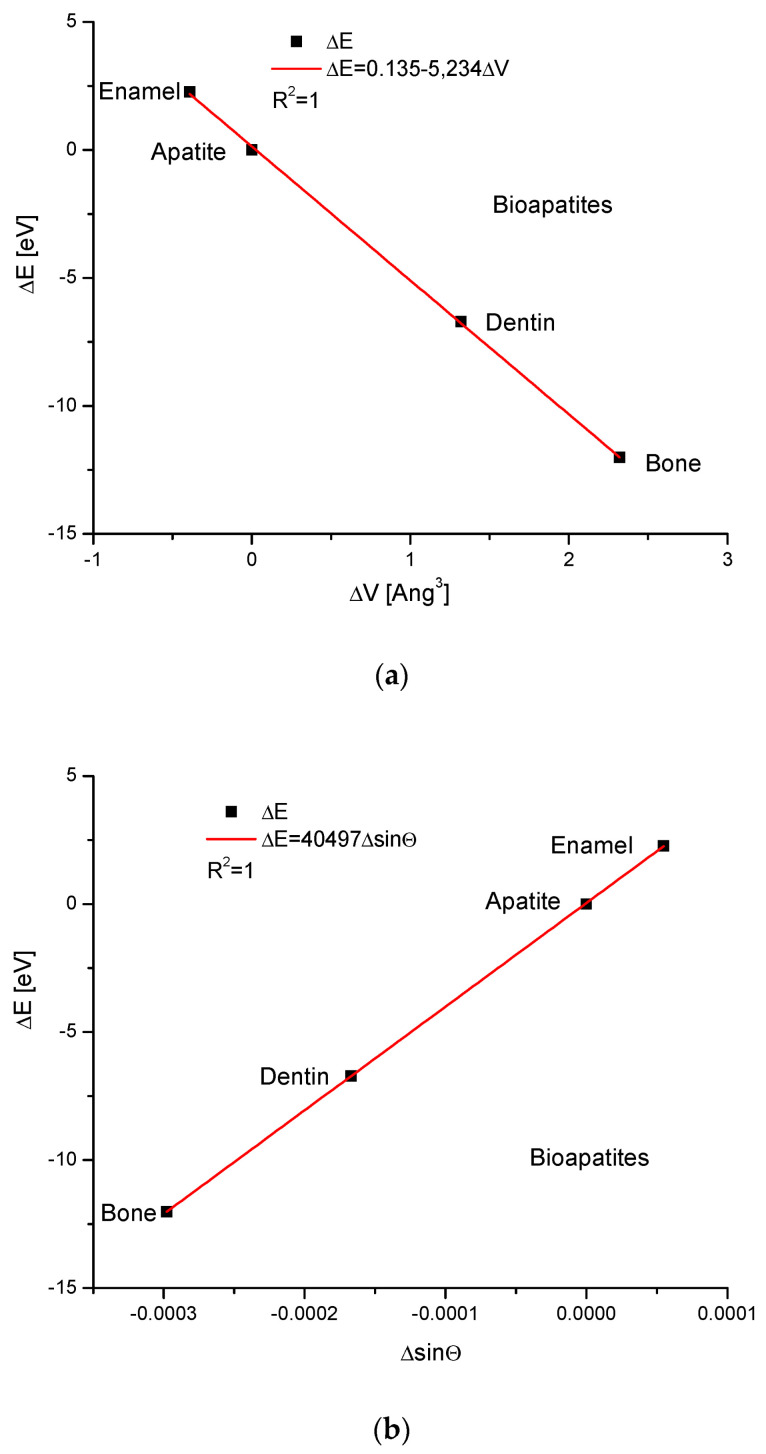

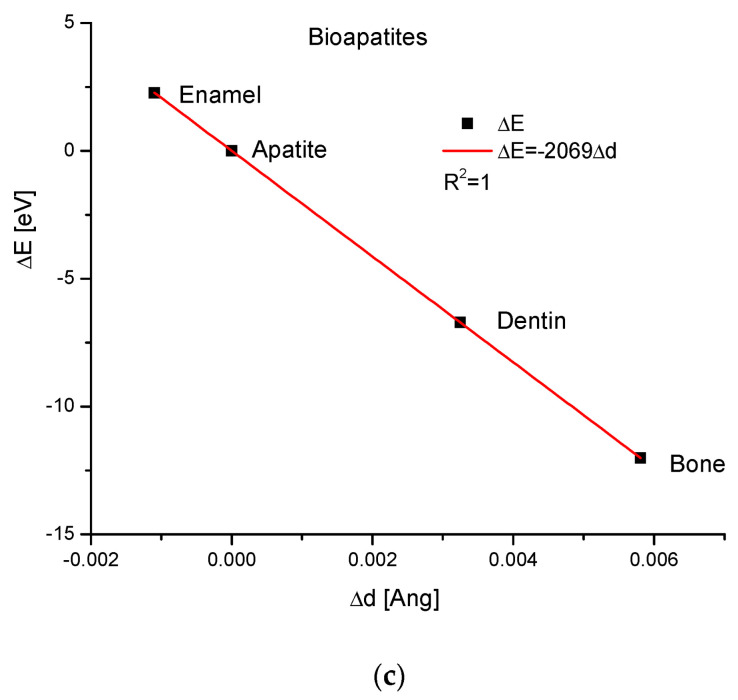
The relationships concerning the energy changes in the bioapatites (**a**) versus the changes in the crystallographic cell volumes (**b**) against the changes in sinΘ values (**c**) in relation to the variations in crystallographic dimension d.

Apatites interest us the most during bio-version. Here, we do not exchange the particular ions, but from this assumption, we consider hydroxyapatite, enamel, bone and dentin as the isomorphic entities. We can observe the dependence of energy changes on the differences in the volumes of the crystallographic cells, differences in the values of the sinus of Θ angle and differences of universal dimension d (Figure 2). All the relationships are rigorously linear. It is worth noting that the change in energy of the enamel is on the opposite side of the series in relation to the pure hydroxyapatite than the positions occupied by the dentin and bone. This crystallographic sequence is different than the chemical sequence established in the paper by Kuczumow et al. [23]. It is interesting that the energy changes occurring due to the changes in both d and sinΘ values go in opposite directions in biomaterials in relation to that which is observed in mineralogical apatites. One can understand it by taking into account the fact that the biological synthesis of bioapatites is deeply antientropic. Besides that, the values of energy changes observed in bioapatites are small. We can say that all exchanges observed in bioapatites are delicate and not as rough as in minerals.

**Figure 3:**
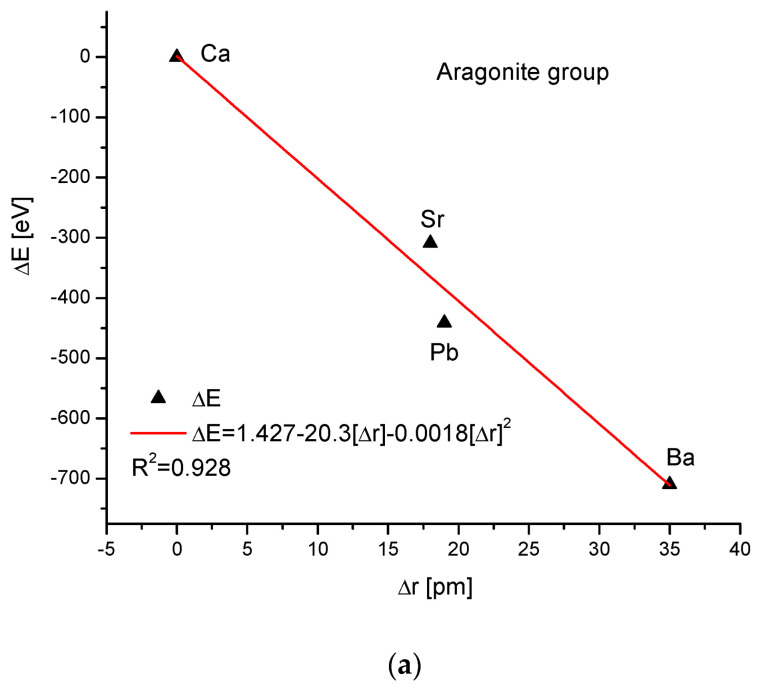

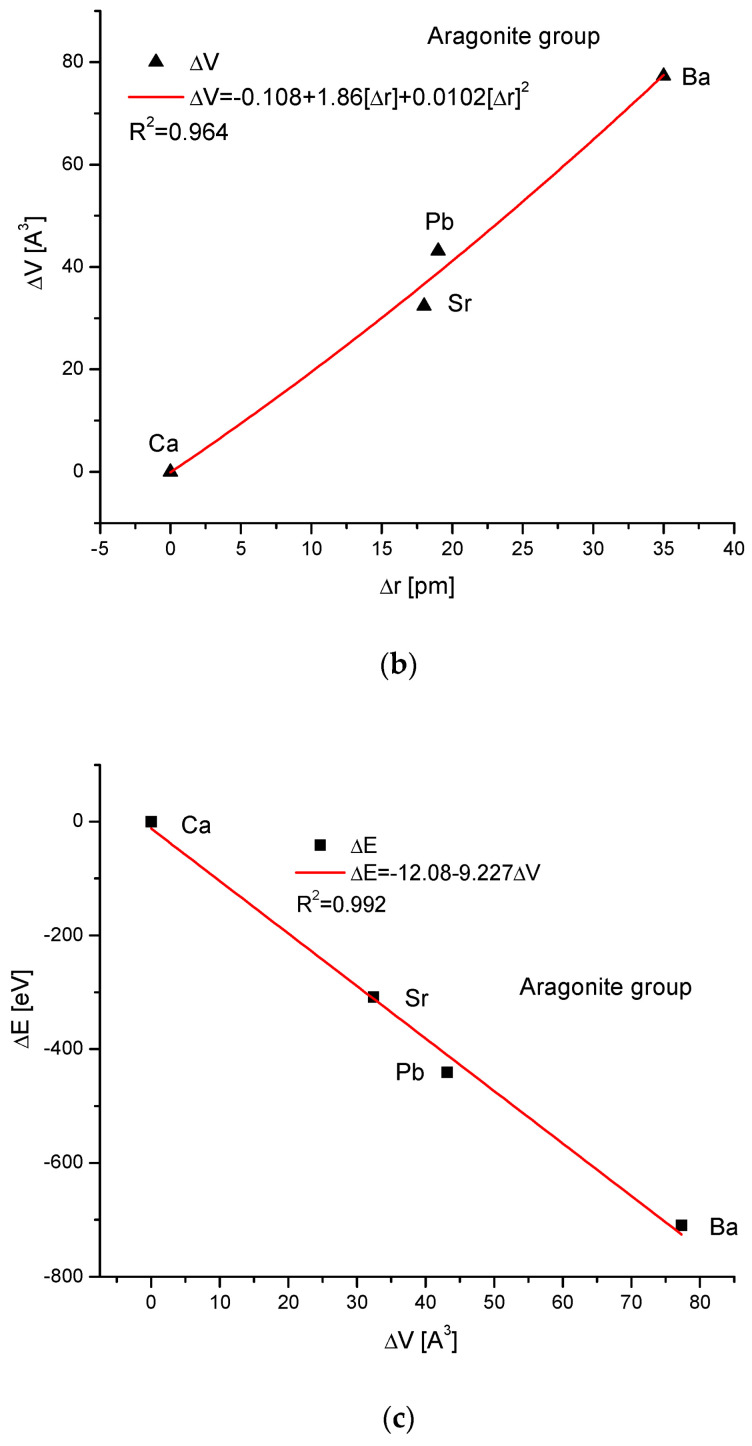

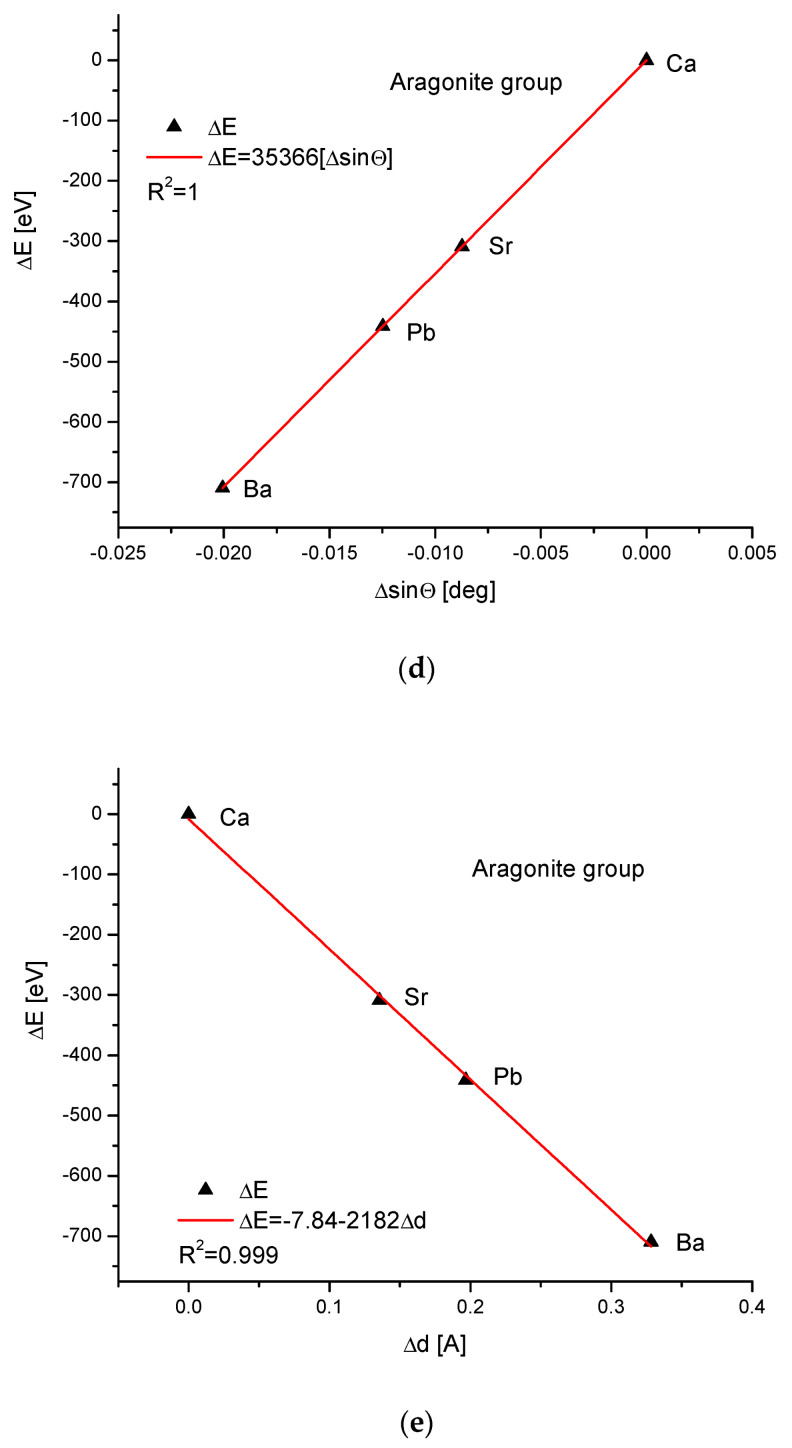
Relationships in the aragonite series. The details of captions are analogous to Figure 1.

As the third isomorphic system, the aragonite series was studied (Figure 3). The relationship of energy change during the complete ion exchange against the change in the ionic radii was nearly linear, with a small correction of the second order. The dependence of the change in cellular volume versus ionic radii was parabolic, where the second-order correction was small. However, the energy demanded for the change in the volume of the crystallographic cell is a linear function. The energy difference as related to the change in sin of Θ angle from diffraction measurements was functionally linear. Finally, the variation in ΔE against the changes in Δd was rigorously linear.

**Figure 4:**
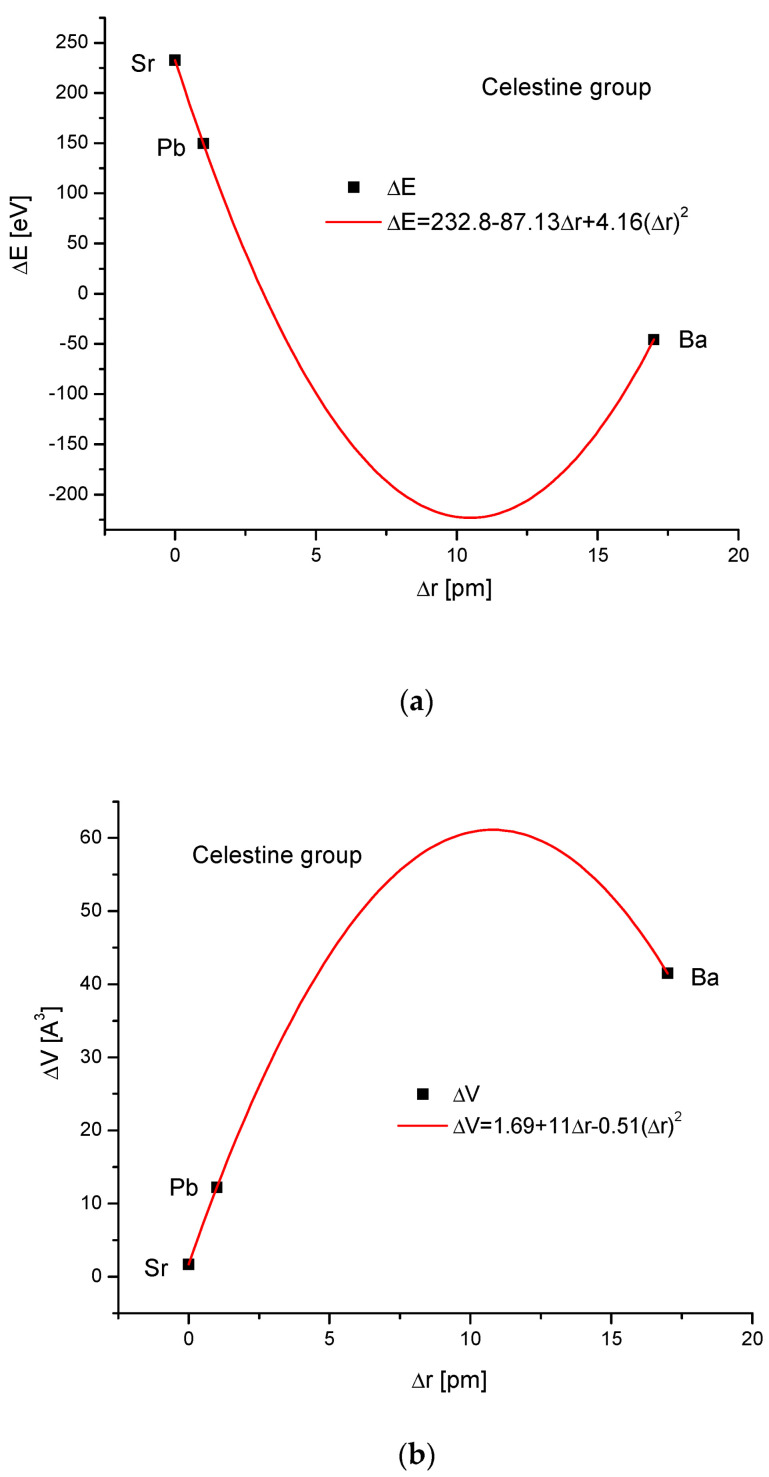

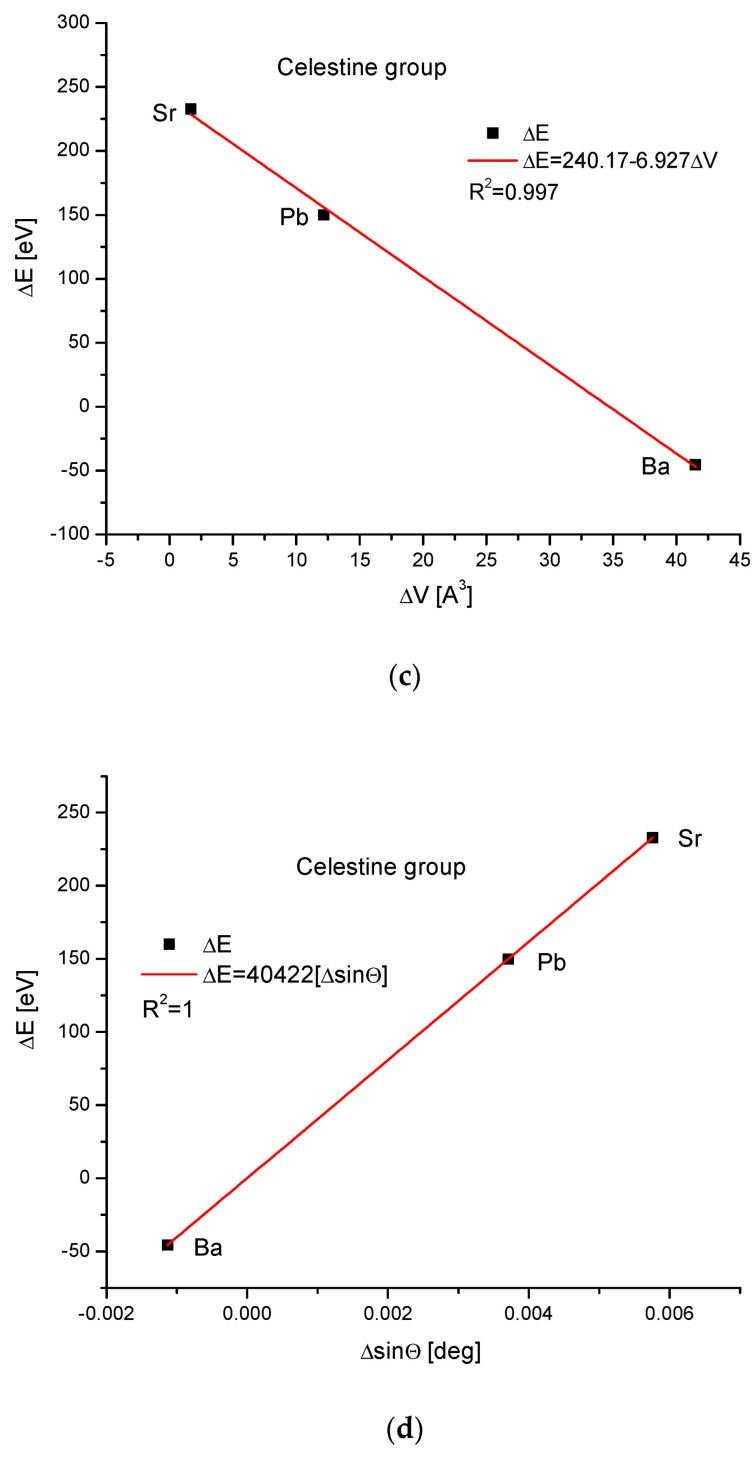

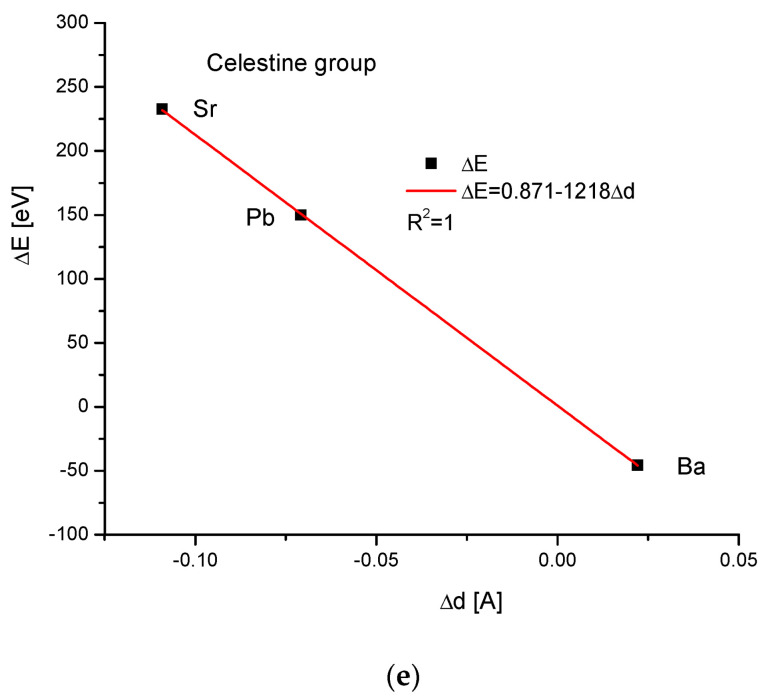
Relationships in the celestine series. The details are analogous to Figure 1.

The celestine group was considered in the same way. Figure 4a,b present clearly paraboloidal dependencies of ΔE on Δr and ΔV on Δr. Here, some problems arrived. The number of members of the celestine series was too small to determine the coefficient of determination for the square-order function. Nevertheless, such a function was applied since it covered all the available points well. Moreover, in other cases, it functioned well for a greater number of points (see Figure 1b and Figure 3b), and we used the analogy principle. The energy necessary for a crystallographic volume increase was presented by the linear function (Figure 4c). The relationship between the energy change and sinΘ was linear again, similarly to the relationship between the energy change and Δd (Figure 4d,e, respectively).

**Figure 5:**
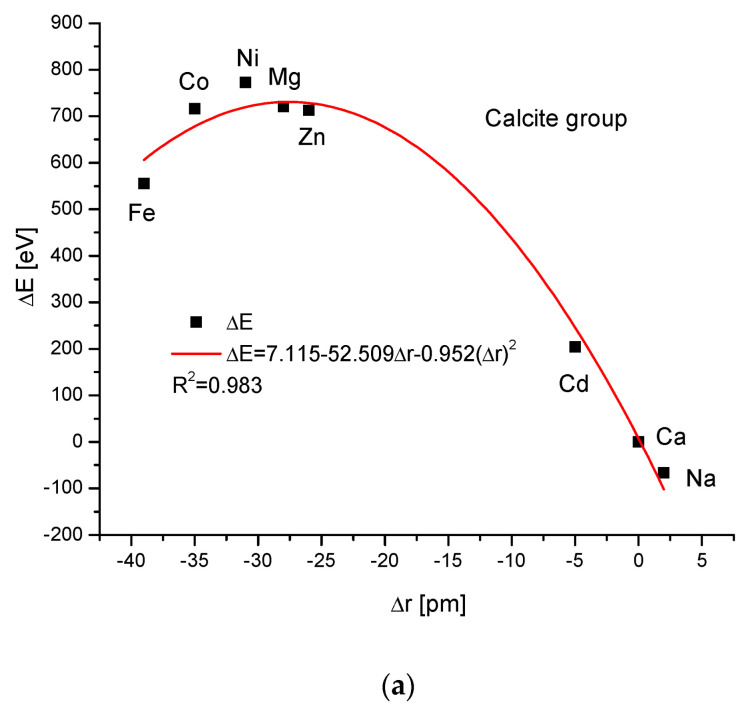

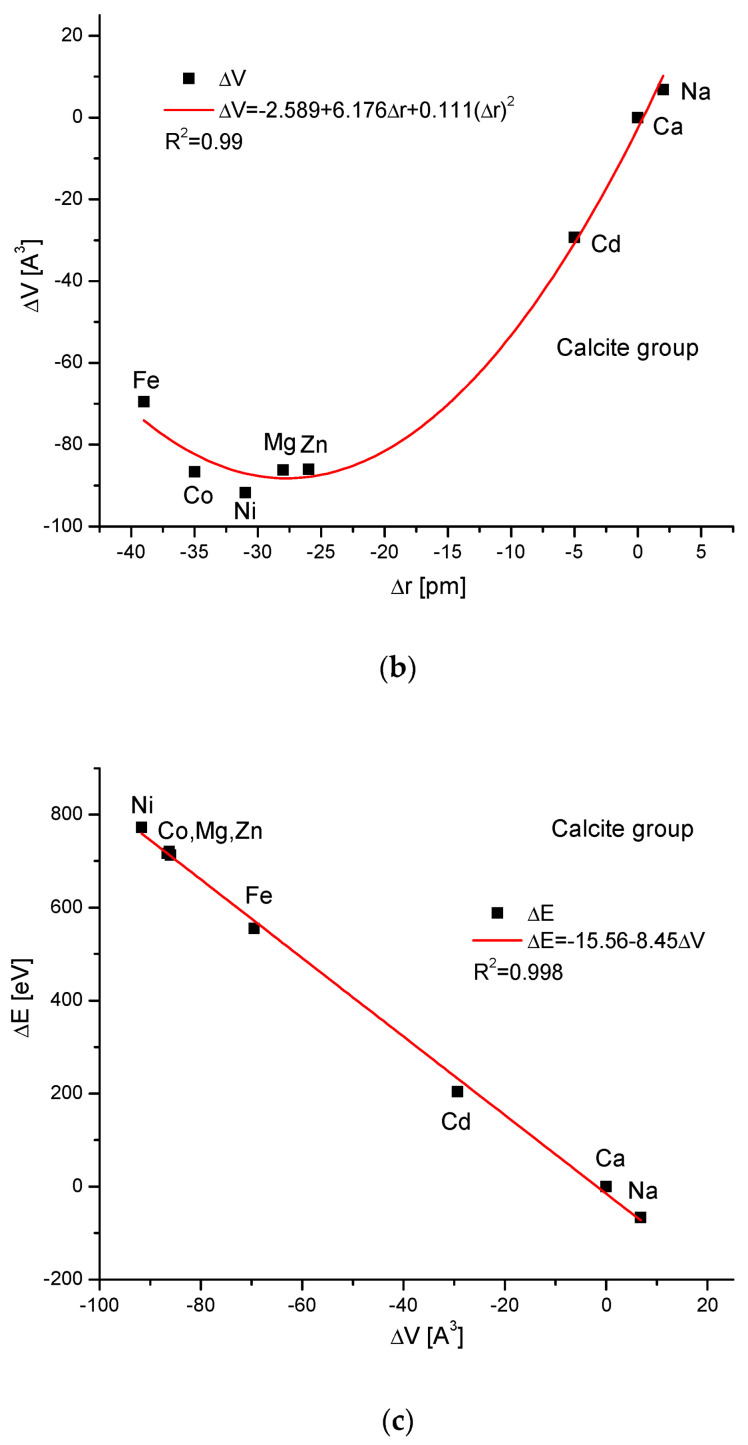

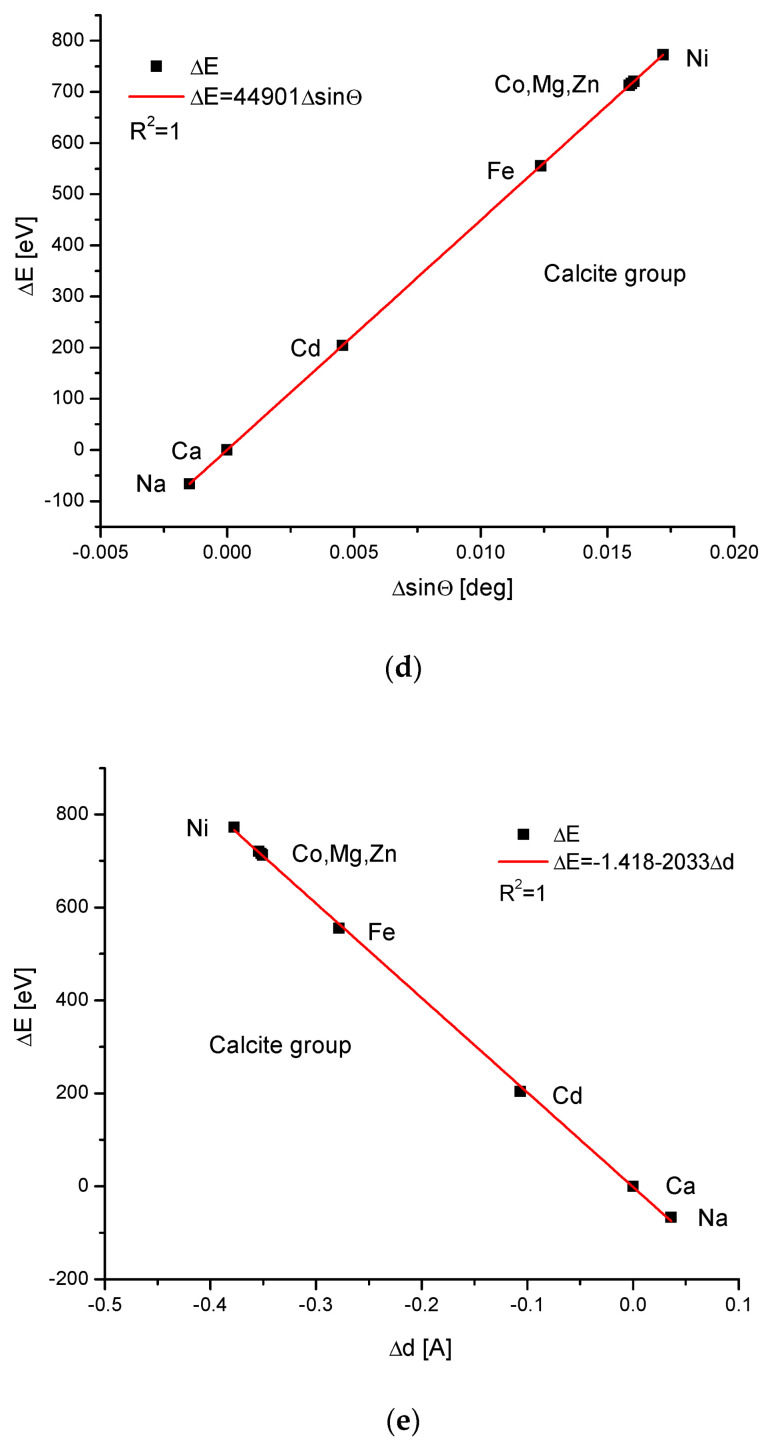
Relationships in calcite series. The details are analogous to Figure 1.

The calcite group is more numerous. Figure 5a,b show, as in the previous case, clear paraboloidal dependencies. The energy joined with the crystallographic cell volume variability is described by a linear function (Figure 5c). Figure 5d,e present once again the functional linear relationships between the changes in energies and changes in sinΘ and d values, respectively. The calcite system has great mineralogical and biological meanings, similarly to the earlier described apatite system.

**Figure 6:**
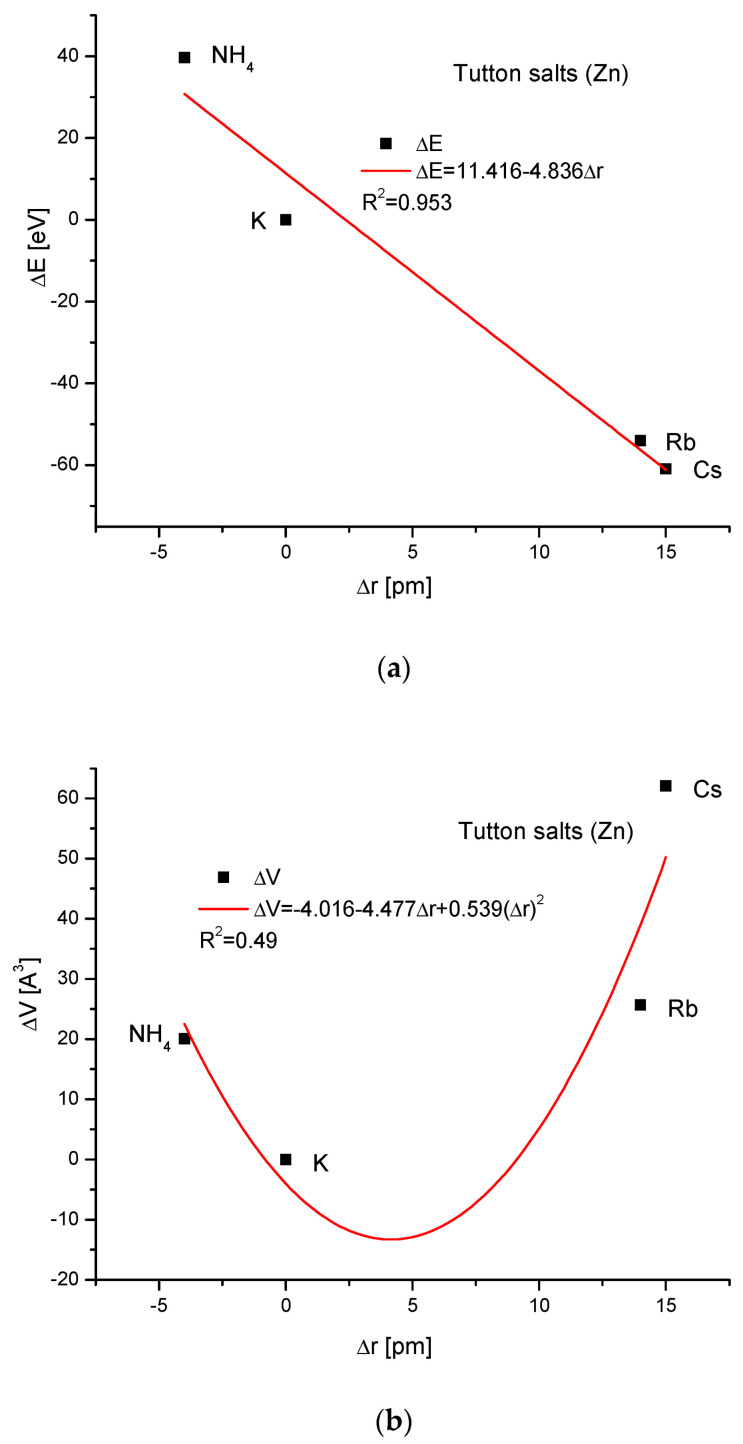

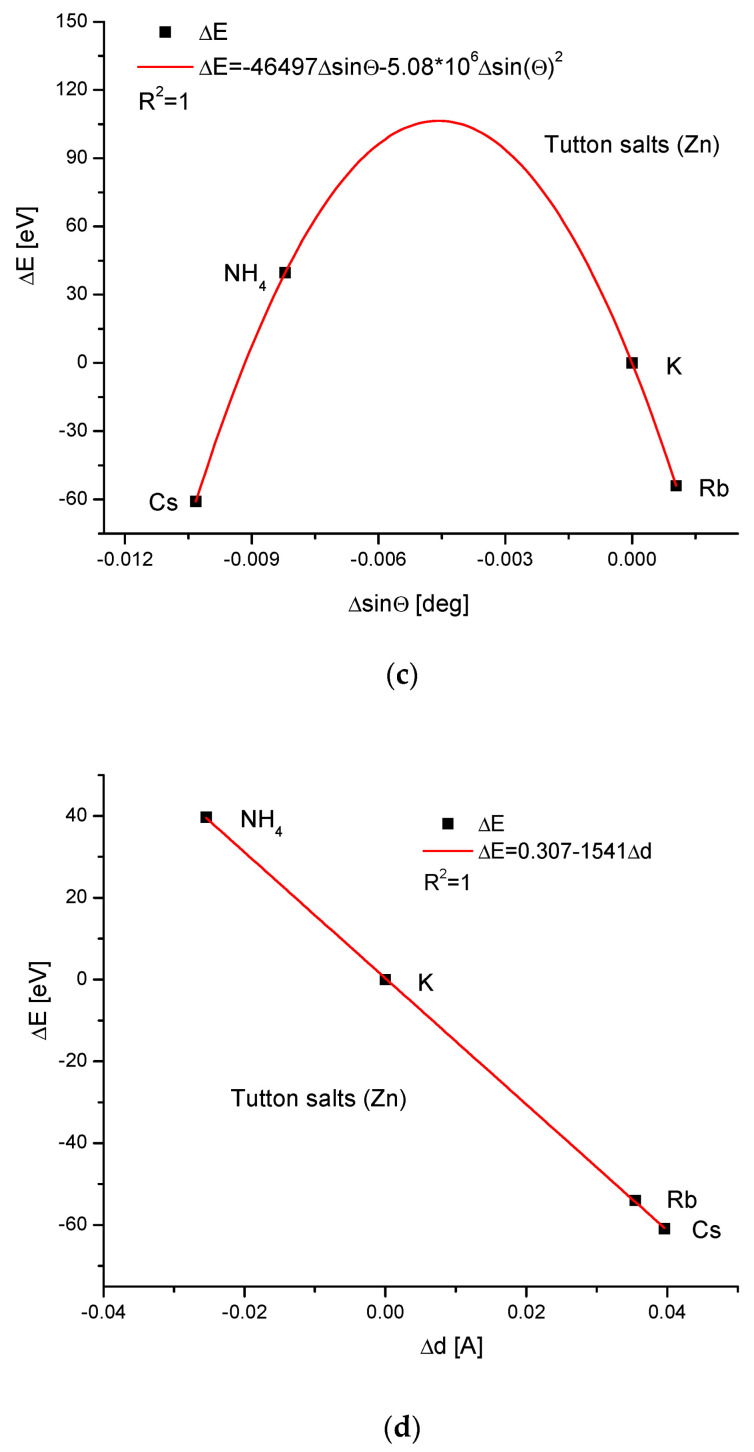
Relationships in Zn Tutton’s series. The details are analogous to Figure 1, except for the missing dependence of energy changes on the changes in the crystallographic cell volume.

Next, the Zn Tutton’s salts are considered (Figure 6). We have to reject the thallium version of this compound (it concerns all Tutton’s salts) since it introduces disorder in our trial of understanding the isomorphic compounds. The relationships between energy changes and ionic radius growth and the essential change in dimension d are linear. The couplings of cell volume changes with the variability of the ionic radius and energy changes with ΔsinΘ, respectively, are handsomely parabolic. The relationship between the energy change and the crystallographic cell volume change was impossible to establish. 

If we select from the different Tutton’s salts—those in which we decide to have constant contents of potassium—we form in that way the K Tutton’s salt series K_2_[Me(H_2_O)_6_](SO_4_)_2_. Here, the relationship of energy changes on the variability of ionic radii is more complicated (third-order polynomial; nevertheless, a perfect one). The relationships between the cell volume and ionic radius changes, and energy and ΔsinΘ changes are parabolic ones. Finally, we go to our general result, i.e., the rigid linear interrelationship between energy and dimension d changes. 

For the Cu version of Tutton’s salts, we managed to establish only the third-order polynomial relationship between the energy difference and the change in ionic radii, as for K Tutton’s salts and, as usual, the rigid linear dependence of energy changes and changes in dimension d. 

## 3. Discussion

The results shown in this contribution are very impressive and testify that the formation of isomorphic compounds obeys rigorous rules, although it is found to be valid for relatively simple compounds. Potentially, starting from estimating the relationship between the ionic radius change and the change in the volume of crystallographic cells is the easiest way for consideration. This relationship is not dependent on our calculations in this contribution. It results immediately from our knowledge of tabularized ionic radii and simple geometrical formulae concerning the geometrical solids (see All Figures, versions b). Except for the case of aragonite series, all other dependencies were the parabolic functions. A similar tendency can be observed in the figures presenting the relationships between the change in the energy of ion exchanges and the differences in ionic radii. The change in energy due to the difference in ionic radii between the changed and changing ions is sometimes simply linear (apatite, aragonite and Zn Tutton’s salt groups), parabolic (celestine and calcite groups) or the third-order polynomials (Cu and K Tutton’s salts). It looks reasonable, since one should intuitively expect that the change in the energy of exchanges should be dependent on the cross-section of entering ions, and that this is the polynomial function of ionic radii. Moreover, the relationships are very simple ones, being polynomials from the first to the third order. Another important type of relationship is the one between the energy necessary for changing the volume of crystallographic cells (all Figures, versions c). They all are linear functions, except for all Tutton’s salts, for which the relationships were found impossible to be regularly described. A somewhat similar situation is the dependency of energy changes on the variability of the sinus Θ angle, which is coupled with the momentum transfer. All the relationships are strictly linear, except the Zn and K Tutton’s salts, where the equations belong to parabolic functions, while for Cu Tutton’s salt, the correlation cannot be determined.

Even more interesting are the relationships between the change in energy of ion exchanges and the differences in the value of universal crystallographic parameter d (last figures in each category of compounds). These regular dependencies are strictly linear, and the coefficients of determination R^2^ are so close to 1 that we can put the functional relationship as:

ΔE = k_1_ + k_2_ × Δd (13)

All the studied cases obeyed the above formulae. Our findings could probably be expanded based on the new kinds of interesting isomorphic compounds, such as covalent organic frameworks (COFs) [24].

If we resign from considering Tutton’s salts, we can add the two next equations to Equation (13):

ΔE = k_3_ × ΔsinΘ (14)

and

**Figure 7:**
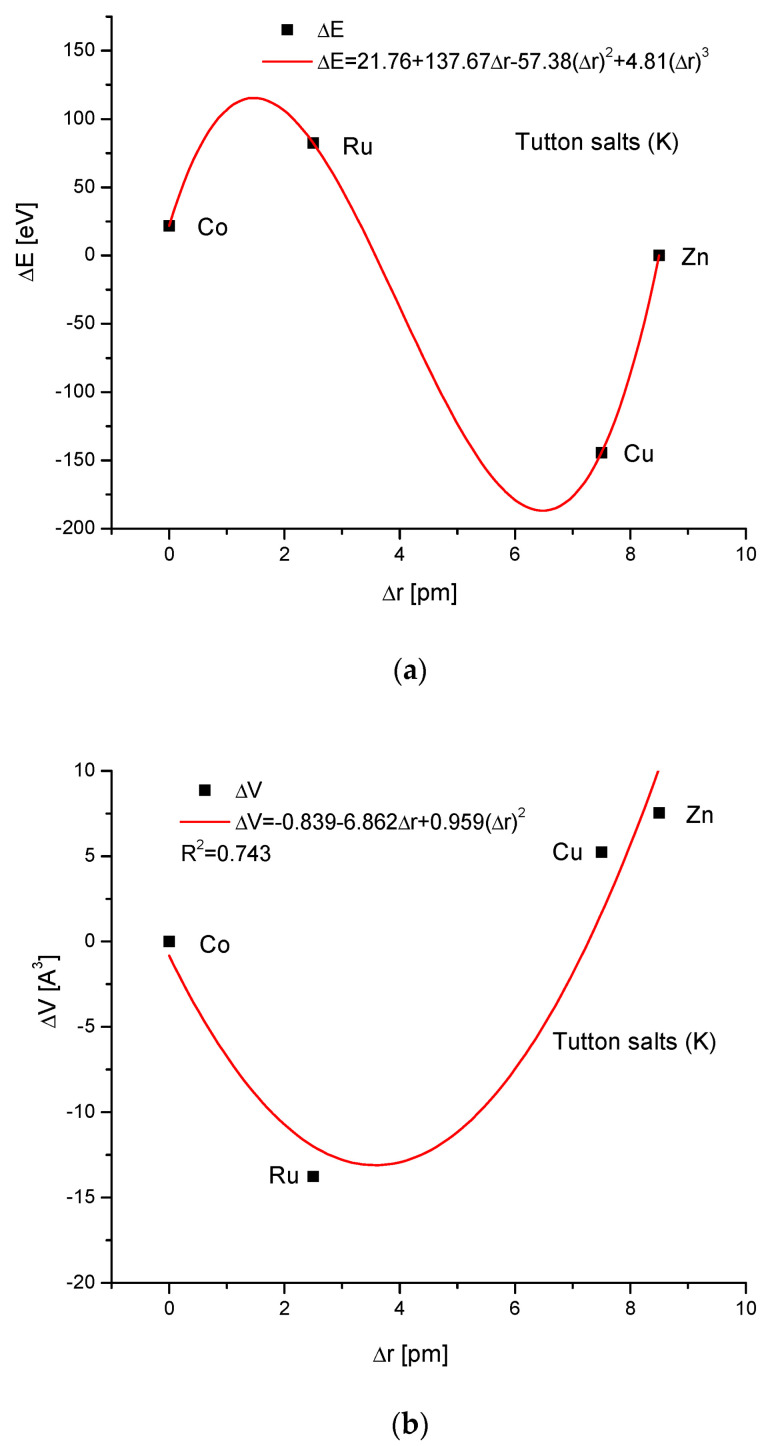

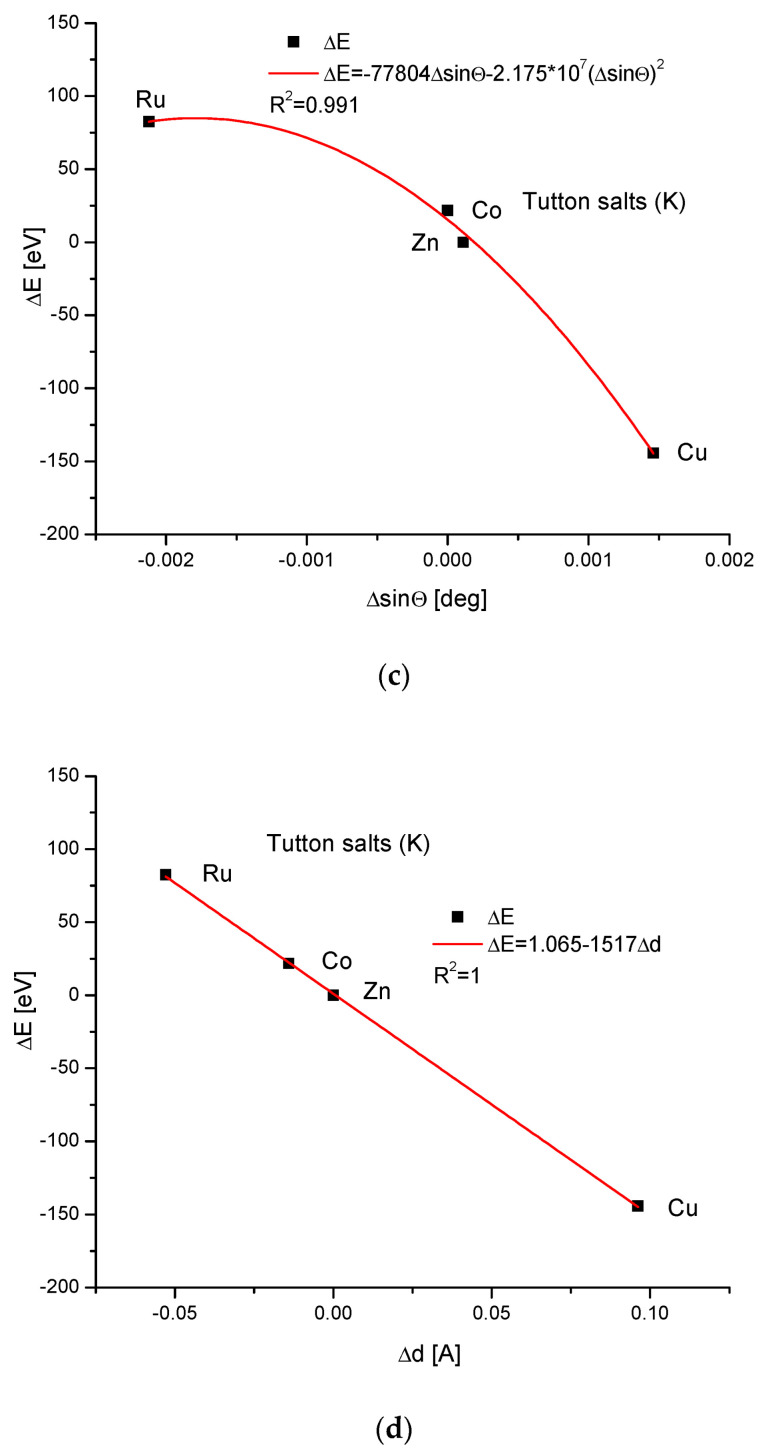
Relationships in K Tutton’s series. The details are analogous to Figure 1.

**Figure 8:**
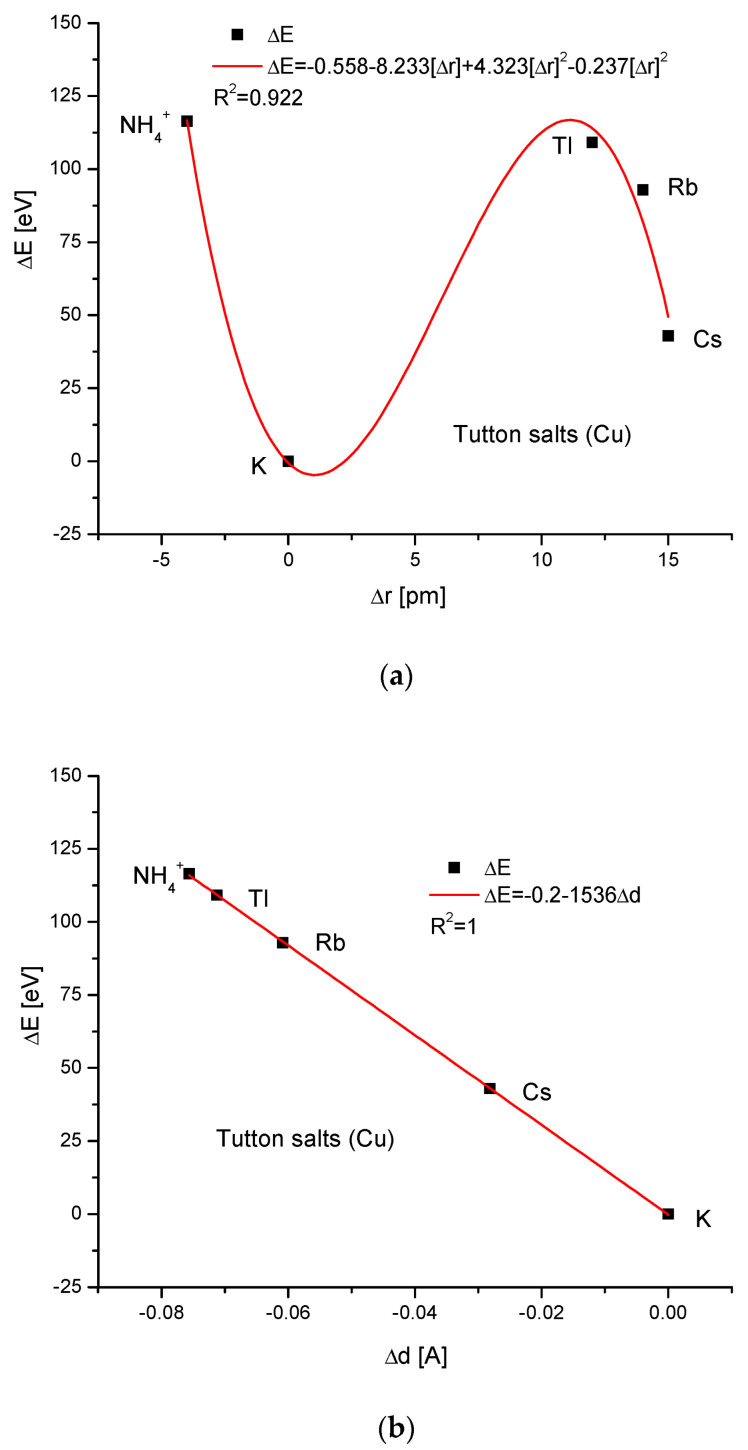
Scarce description of Cu Tutton’s salt: (**a**) the relationship between energy changes and the radii of ions entering the crystallographic network; (**b**) the dependence of energy changes on crystallographic dimension d variability.

ΔE = k_4_+ k_5_ × ΔV (15)

where designations k are constant. The three equations describe the quantitative relationships in the simple isomorphic compounds. For more complicated cases of Tutton’s salts, only Equation (13) is valid, which otherwise seems to be the universal relationship for the isomorphic compounds. Tutton’s salts seem to be an exception, since they can only be described by Equation (13), not by the two further ones. This is probably caused by the steric effects—we can see how complicated the dependencies are between the energies of ion exchanges and ionic radii in Figure 7a and Figure 8a in comparison with all the other cases. This steric effect is clear, even taking into account the small difference in ionic radii. The next reason can be derived from the different electronegativities and solubilities between Cu and Zn. Finally, the momentum transfer in Figure 6c and Figure 7c is different than in previous cases. 

The coupling between Δd, ΔV and ΔsinΘ is shown in Appendix B.

## 4. Materials and Methods

We used the available data concerning the apatite [14], calcite [25], aragonite [26], and celestine groups [27,28] and selected Tutton’s salts Me_2_[Cu(H_2_O)_6_](SO_4_)_2_ [29,30,31], also with Zn [32] and K. The data involved the relevant crystallographic length dimensions and angles for the compounds under study and the information about the kind of crystallographic class. They were collected using independent X-ray diffraction (XRD) measurements by different authors. The studied substances were either natural or synthetized compounds.

The following substances are the members of:

- The apatite group: apatites derived from the hydroxyapatite 3Ca_3_(PO_4_)_2_*Ca(OH)_2_, with cationic substitutions of Mg, Cd, Pb, Sr and Ba^16^.
- The calcite group: magnesite MgCO_3,_ calcite CaCO_3_, siderite FeCO_3_, spherocobaltite CoCO_3_, gaspeite NiCO_3_, smithsonite ZnCO_3_, otavite CdCO_3_ and nitratine NaNO_3_. Rhodocrosite MnCO_3_ was rejected, probably due to the variable valence state of Mn and the different environments of ions.
- The aragonite group: aragonite CaCO_3_, strontianite SrCO_3_, witherite BaCO_3_, and cerussite PbCO_3_.
- The celestine group: anhydrite CaSO_4_, SrSO_4_, barite BaSO_4_, and anglesite PbSO_4_.
- Zn Tutton’s Me_2_[Zn(H_2_O)_6_](SO_4_)_2_ salt with substitutions with NH_4_^+^, K, Rb, and Cs.
- Cu Tutton’s Me_2_[Cu(H_2_O)_6_](SO_4_)_2_ salt with Me: NH_4_^+^, K, Rb, and Cs.
- K Tutton’s K_2_[Me(H_2_O)_6_](SO_4_)_2_ with Me: Zn, Co, Cu, and Ru.

Tl containing Tutton’s salts, which were the members of the relevant series, were excluded from the considerations, since this element introduced serious disturbances in our calculations.

## 5. Conclusions

A couple of series of popular isomorphic substances were studied. The changes in energies during the transitions from one member of the series to the consecutive member were analyzed. In nearly all the series, very clear relationships occurred. All the relationships were the polynomials of the first and second order; in the case of Zn and K Tutton’s series only, the dependence between ΔE and the change in ionic radii demanded involving a third-order term. Without any exceptions, all the relationships between the change in energy and in universal crystallographic parameter d (configuration (111)) were rigorously linear; this is the most important characteristic of the studied isomorphic compounds. It testifies that the growth of energy of isomorphic ion exchanges is functionally related with the change in d. For all the cases except Tutton’s salts, the linear relationships were found in the systems: ΔE − Δd; ΔE − ΔV; ΔE − ΔsinΘ; ΔV − Δd; ΔV − ΔsinΘ and Δd − ΔsinΘ. 

## Figures and Tables

**Figure A1:**
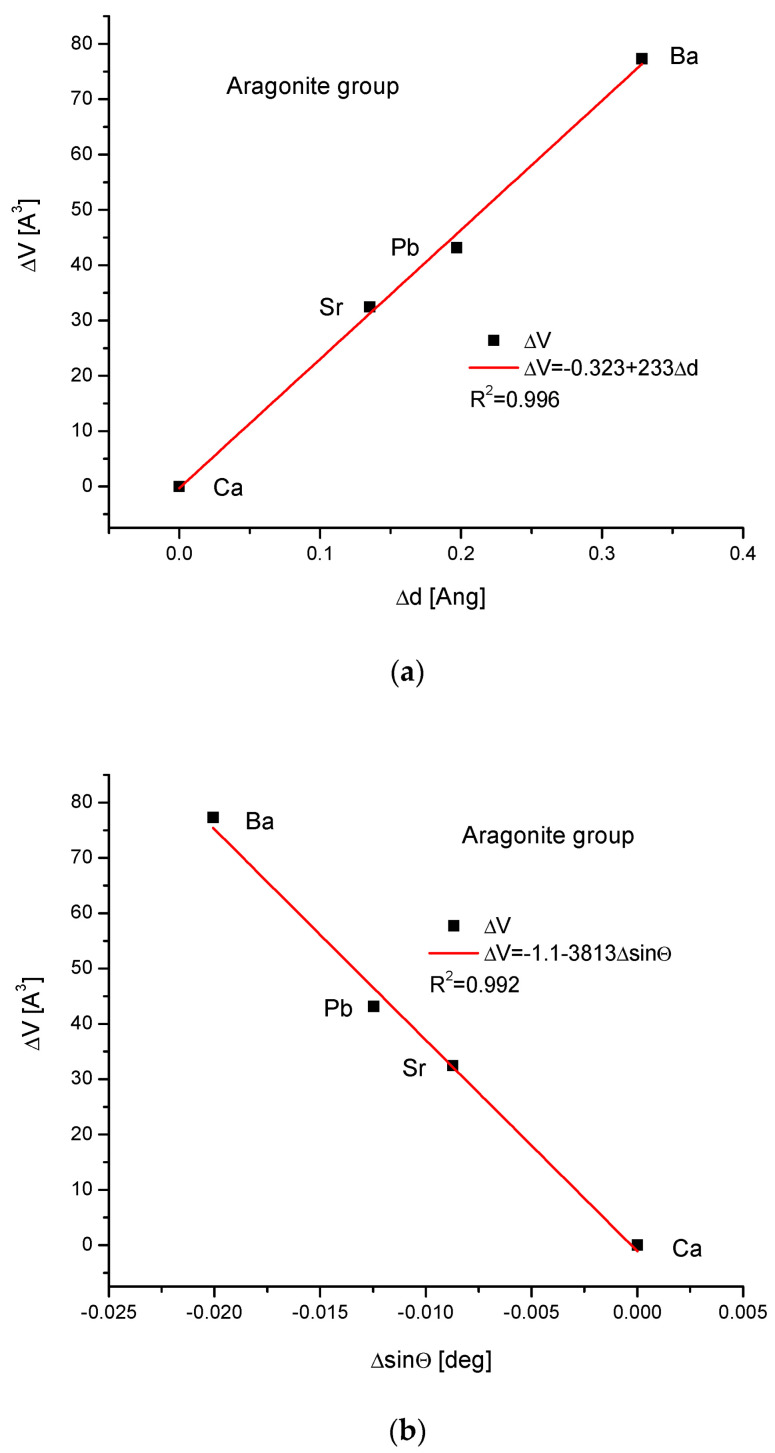

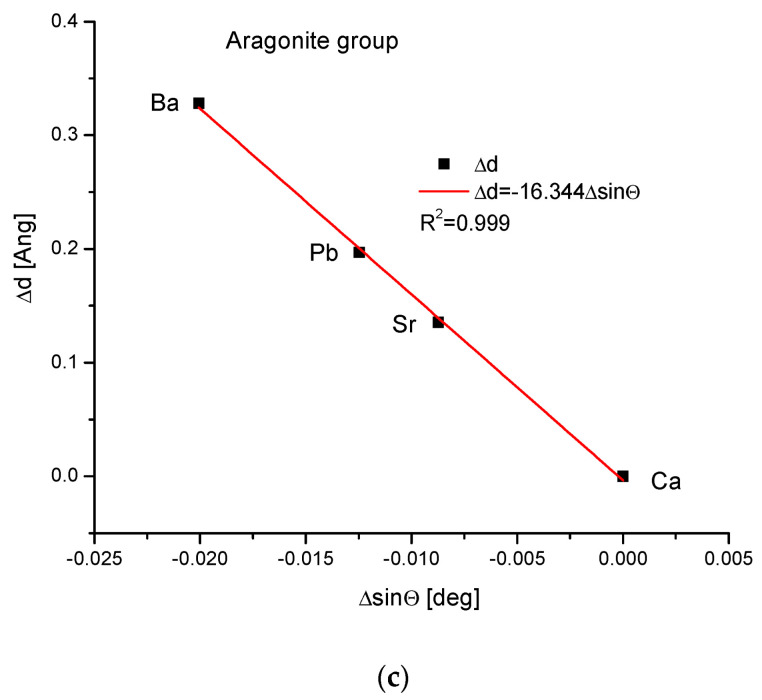
(**a**) The connection between the growth of volume of crystallographic cells and universal Bragg’s dimension d; (**b**) the connection between the volume and sinus of diffraction angle Θ; (**c**) the connection between Δd and ΔsinΘ.

## Data Availability

No new data were created; the presented mathematical models were based on previously published manuscripts which were referenced in the text.

## Appendix A

We can use Equation (1) for the calculations, e.g., the energy difference between the extreme members of calcite series. It demands the knowledge of d parameters for nitratine NaNO_3_ and gaspeite NiCO_3_: 4.02839Å and 4.34263, respectively, and of sinΘ: 0.19143 and 0.17758. After calculations according to Equations (2)–(6), one can obtain: −627.05 eV; −627.05 eV; −627.16 eV; −627.05 eV; −627,34 eV. The deviations are of the order of 0.3 eV at the level ~600 eV.

## Appendix B

Here, we can observe the additional connections between Δd, ΔV and ΔsinΘ. Equations (13)–(15) in the main text suggest that there are very strong and rigorous links among those parameters. Indeed, all the relationships are linear, with the coefficients of determination at level 1. It puts the question forward: are the dependencies, in reality, the true functional equations?
